# Diagnostic accuracy of palpation versus ultrasound-guided fine needle aspiration biopsy for diagnosis of malignancy in thyroid nodules: a systematic review and meta-analysis

**DOI:** 10.1186/s12902-022-01085-5

**Published:** 2022-07-17

**Authors:** Tri Juli Edi Tarigan, Budiman Syaeful Anwar, Robert Sinto, Wismandari Wisnu

**Affiliations:** 1 Division of Endocrinology, Metabolism and Diabetes, Department of Internal Medicine, Dr. Cipto Mangunkusumo National General Hospital, Faculty of Medicine Universitas Indonesia, Jakarta, Indonesia; 2 Metabolic, Cardiovascular and Aging Cluster, The Indonesian Medical Education and Research Institute, Faculty of Medicine Universitas Indonesia, Jakarta, Indonesia; 3 Department of Internal Medicine, Dr. Cipto Mangunkusumo National General Hospital, Faculty of Medicine Universitas Indonesia, Jakarta, Indonesia; 4 Division of Infection and Tropical Disease, Department of Internal Medicine, Dr. Cipto Mangunkusumo National Referral Hospital, Faculty of Medicine, Universitas Indonesia, Jakarta, Indonesia

**Keywords:** Fine-needle aspiration biopsy, Palpable versus USG guided, Thyroid nodules

## Abstract

Thyroid nodule is a common health problem in endocrinology. Thyroid fine-needle aspiration biopsy (FNAB) cytology performed by palpation guided FNAB (PGFNAB) and ultrasound-guided FNAB (USGFNAB) are the preferred examinations for the diagnosis of thyroid cancer and part of the integration of the current thyroid nodule assessment. Although studies have shown USGFNAB to be more accurate than PGFNAB, inconsistencies from several studies and clinical guidelines still exist.

The purpose of this study is to compare the diagnostic accuracy of Palpation versus Ultrasound-Guided Fine Needle Aspiration Biopsy in diagnosing malignancy of thyroid nodules.

The systematic review and meta-analysis were prepared based on the PRISMA standards. Literature searches were carried out on three online databases (Pubmed/MEDLINE, Embase, and Proquest) and grey literatures. Data extraction was carried out manually from various studies that met the eligibility, followed by analysis to obtain pooled data on sensitivity, specificity, Diagnostic Odds Ratio (DOR) and Area Under Curve (AUC), and the comparison of the two methods.

Total of 2517 articles were obtained, with 11 studies were included in this systematic review. The total sample was 2382, including 1128 subjects using PGFNAB and 1254 subjects using USGFNAB. The risk of bias was assessed using QUADAS-2 with mild-moderate results. The results of sensitivity, specificity, AUC and DOR in diagnosing thyroid nodules using PGFNAB were 76% (95% CI, 49–89%), 77% (95% CI, 56–95%), 0.827 and 11.6 (95% CI, 6–21) respectively. The results of sensitivity, specificity, AUC and DOR in diagnosing thyroid nodules using USGFNAB were 90% (95% CI, 81–95%), 80% (95% CI, 66–89%), 0.92 and 40 (95% CI, 23–69), respectively the results of the comparison test between PGFNAB and USGFNAB; Tsens USGFNAB of 0.99 (*p* = 0.023), AUC difference test of 0.093 (*p* = 0.000023).

The diagnostic accuracy of USGFNAB is higher than PGFNAB in diagnosing malignancy of thyroid nodules. If it is accessible, the author recommends using USGFNAB as a diagnostic tool for thyroid nodules.

## Introduction

Thyroid nodule, either it is solitary or multiple, is a common endocrinology problem in daily clinical practice [1]. On physical examination, thyroid nodules are detected in about 5–7% of the adult population [2, 3]. As the use of ultrasonography (USG) has increased, there has been an increase in the incidence of thyroid nodules by 19–68% in previously undetected cases [3, 4].

The gold standard to diagnose thyroid nodules is the histopathological finding from surgical biopsy. However, the examination is invasive, expensive, and not easy to perform because it requires a long process from clinical evaluation to indicate surgery for the patient [2, 4]. An alternative examinations that can be done is the fine needle aspiration biopsy (FNAB). FNAB examination is widely accepted as an excellent diagnostic tool for evaluating thyroid nodules because it is sensitive, specific, cost-effective, and low risk for complications [3, 5]. Furthermore, a study by Ospina et al. reported that FNAB had a sensitivity ranging from 57 to 93% with a false positive of about 3% and a false negative rate of 5% [4]. There are two methods of FNAB on palpable thyroid nodules, performed with a palpation-guided fine-needle aspiration biopsy (PGFNAB) or ultrasound-guided fine-needle aspiration biopsy (USGFNAB) [1].

Previous researchers have carried out systematic reviews and meta-analyzes of PGFNAB and USGFNAB. A study by Matz et al., which was carried out in 1108 patients with PGFNAB and 1197 patients with USGFNAB, indicated that USGFNAB had a higher diagnostic accuracy but with a lower inadequate sample rate than PGFNAB [6]. On the contrary, according to a study conducted by Choong et al. in 2018 on 2322 patients who underwent FNAB, the same number of indeterminants and false negatives in both the PGFNAB and USGFNAB groups were obtained [7]. In a meta-analysis conducted by Ospina et al. of 32 studies examining the diagnostic accuracy of USGFNAB proved that USGFNAB had a moderate risk of bias with results that were not always accurate and heterogeneous [4]. In a study by Taha et al. which included 1174 subjects in 2020 and divided subjects into the USGFNAB (33.4%) and PGFNAB (48.6%) groups, found that diagnostic accuracy was not much different between USGFNAB and PGFNAB. The proportion of malignant case findings was higher in the USGFNAB group than in PGFNAB (8.9 vs 6.4%). These results were confirmed by postoperative histopathological examination (p 0.95) [5].

Several organizations related to the thyroid nodule diagnosis approach have different recommendations for the use of USGFNAB and PGFNAB. The American Thyroid Association (ATA), US National Cancer Institute (NCI), the British Thyroid Association (BTA), the American Association of Clinical Endocrinologists (AACE), the Associazione Medici Endocrinology (AME), the European Thyroid Association (ETA) recommend that non-palpable, hard palpable, or partially cystic thyroid nodules should be evaluated by ultrasound. Meanwhile, for palpable thyroid nodules, AACE, AME, and ETA still suggest doing USGFNAB, while others may use PGFNAB or USGFNAB [8].

Therefore, through this meta-analysis, a systematic review was conducted because of the inconsistence found in previous studies and existing guidelines (Table 1). Moreover, several recent studies are expected to obtain results to better describe this current situation.

**Table 1:** Inadequacy rate of PGFNAB and USGFNAB in each study

Researcher & Year	Number of nodules	PGFNAB	USGFNAB	Source
Takashima 1994 [9]	330	4% (10/268)	19% (12/62)	23
Danese 1998 [10]	9683	9% (433/4986)	3.5% (167/4697)	15
Hatada 1998 [11]	166	30% (28/94)	17% (12/72)	17
Carmeci 1998 [12]	497	16% (60/370)	7% (9/127)	18
Goudy 2005 [13]	89	11.5% (9/78)	0% (0/11)	16
Cesur 2006 [14]	285	32% (92/285)	21.5% (61/285)	13
Izquierdo 2006 [15]	376	11% (19/170)	7% (16/225)	14
Can 2008 [16]	386	27% (55/202)	13% (23/184)	19
Krishnappa 2013 [17]	91	11% (6/91)	2% (2/91)	20
Guo 2015 [18]	489	2% (2/101)	2% (8/388)	12
Sharma 2017 [19]	410	15% (36/237)	0.5% (1/173)	21
Choong 2018 [7]	2322	4.5% (50/1123)	5% (55/1199)	7
Taha 2020 [5]	962	17% (98/570)	13% (51/392)	5

## Materials and methods

### Literature search

The literature search was performed on PubMed/ MEDLINE, Embase, and ProQuest. The search had been carried out with keywords based on MESH terms and their synonyms and with the use of Boolean operator assistance. Moreover, the author searched for other sources by searching the registry for observational studies or manuals for relevant studies or in the form of grey literature such as abstracts of symposiums/ conferences/ proceeding books/ theses/ dissertations or through the portal on the Garba Digital Referral (GARUDA) page – a local online database. Keywords used for the search and snowball search can be seen on Tables 2 and 3. Also, the author tried to contact the lead author of the PGFNAB versus USGFNAB diagnostic accuracy articles via correspondence email to search for studies that the authors may not have found. The literature search was conducted until November 25, 2020. This attempt was made to ensure that all relevant studies could be included in this systematic review.

**Table 2:** Query MesH terms

“thyroid nodules”[Title/Abstract] OR “thyroid neoplasms”[MeSH Terms] OR “Goiter”[Title/Abstract] OR “Struma”[Title/Abstract] OR “thyroid cancers”[Title/Abstract]	
AND	
“biopsy, fine needle”[MeSH Terms] OR “fine needle aspiration biopsy”[Title/Abstract] OR “FNAB”[Title/Abstract] OR “FNAC”[Title/Abstract] OR ((“biopsi”[All Fields] AND “jarum”[All Fields]) AND “halus”[Title/Abstract]) OR “BAJAH” [Title/Abstract]	
AND	
“ultrasound guided fine needle aspiration biopsy”[Title/Abstract] OR “USGFNAB”[Title/Abstract] OR “Ultrasound”[Title/Abstract] OR “Ultrasonography”[Title/Abstract] OR “US”[Title/Abstract] OR “palpation guided fine needle aspiration biopsy”[Title/Abstract] OR “PGFNAB”[Title/Abstract] OR “Palpate”[Title/Abstract] OR “Palpation”[Title/Abstract]

**Table 3:** The search results from various databases

Database	Keyword	Hit
Pubmed/MEDLINE	(“thyroid nodules”[Title/Abstract] OR “thyroid neoplasms”[MeSH Terms] OR “Goiter”[Title/Abstract] OR “Struma”[Title/Abstract] OR “thyroid cancers”[Title/Abstract]) AND (“biopsy, fine needle”[MeSH Terms] OR “fine needle aspiration biopsy”[Title/Abstract] OR “FNAB”[Title/Abstract] OR “FNAC”[Title/Abstract] OR ((“biopsi”[All Fields] AND “jarum”[All Fields]) AND “halus”[Title/Abstract])) AND (“ultrasound guided fine needle aspiration biopsy”[Title/Abstract] OR “USGFNAB”[Title/Abstract] OR “Ultrasound”[Title/Abstract] OR “Ultrasonography”[Title/Abstract] OR “US”[Title/Abstract] OR (“palpation guided fine needle aspiration biopsy”[Title/Abstract] OR “PGFNAB”[Title/Abstract] OR “Palpate”[Title/Abstract] OR “Palpation”[Title/Abstract]))	2089
Embase	#1 thyroid nodules m_titl, #2 Limit #1 to abstracts #3 thyroid tumor/ #4 goiter m_titl #5 Limit #4 to abstracts #6 thyroid cancers #7 Limit #6 to abstracts #8 Struma m_titl #9 Limit #8 to abstracts #10 Fine needle aspiration bio psy/ #11 Fine needle aspiration biopsy m_titl #12 Limit #11 to abstracts #13 FNAB m_titl #14 Limit #13 to abstracts #15 FNAC m_titl #16 Limit #15 to abstracts #17 biopsi aspirasi jarum halus mp_title #18 ultrasound-guided fine-needle aspiration biopsy m_titl #19 Limit #18 to abstracts #20 USGFNAB m_titl #21 Limit #20 to abstracts #22 ultrasound m_titl #23 Limit #22 to abstracts #24 ultrasonography m_titl #25 Limit #24 to abstracts #26 US m_titl #27 Limit #26 to abstracts #28 palpation guided fine needle aspiration biopsy m_titl #29 Limit #28 to abstracts #30 PGFNAB #31 Limit #30 to abstracts #32 Palpate m_titl #33 Limit #32 to abstracts #34 Palpation m_titl #35 Limit #34 to abstracts #36 #1 OR #2 OR #3 OR #4 OR #5 OR #6 OR #7 OR #8 OR # 9 #37 #10 OR #11 OR #12 OR#13 OR #14 OR #15 OR #16 OR #17	46
	#38 #18 OR #19 OR #20 OR #21 OR #22 OR #23 OR #24 OR #25 OR #26 OR #27 OR #28 OR #29 OR #30 OR #31 OR #32 OR #33 OR #34 OR #35 #39 #36 AND #37 AND #38	
Proquest	#1 ab,ti(“thyroid nodules”) OR ab,ti(“thyroid neoplasms”) OR ab,ti(“thyroid cancers”) OR ab,ti(“Goiter”) OR ab,ti(“Struma”) #2 ab,ti(“biopsy, fine needle”) OR ab,ti(“fine-needle aspiration biopsy”) OR ab,ti(“FNAB”) OR ab,ti(“FNAC”) OR ab,ti(“biopsi aspirasi jarum halus”) OR ab,ti(“BAJAH”) #3 ab,ti(“ultrasound-guided fine-needle aspiration biopsy”) OR ab,ti(“USGFNAB”) OR ab,ti(“Ultrasound”) OR ab,ti(“Ultrasonography”) OR ab,ti(“US”) #4 ab,ti(“palpation-guided fine-needle aspiration biopsy”) OR ab,ti(“PGFNAB”) OR ab,ti(“Palpate”) OR ab,ti(“Palpation”) #1 AND #2 AND (#3 OR #4)	1364
*Grey Literature*		38

### Study selection

Two researchers (BSA and TJET) carried out the study selection independently, using guidelines based on predetermined eligibility criteria. The two researchers independently screened titles and abstracts from the search results for the study. Then, each study that was considered to meet the eligibility criteria was read in total. It was also reassessed to meet the eligibility criteria based on a form that had been prepared previously. The assessment of the two studies were hidden from each researcher. If any differences of opinion were to arise between the two researchers, it was resolved by consensus and consultation with a third independent researcher was conducted to determine the final assessment if needed. The level of agreement between researchers was assessed using Cohen’s Kappa statistics. The Covidence software was used to screen titles and abstracts and record all decisions made independently by researchers.

All years publication studies included in this research met the inclusion criteria such as (a) diagnostic studies; (b) subjects of all ages; (c) quantitative data from the results to make the 2 × 2 table to obtain the true positive, true negative, false positive, and false negative; (d) the palpation-guided thyroid nodule compared with histopathology of surgery results; (e) ultrasound-guided thyroid nodule compared with the histopathology of surgery results; (f) the compared accuracy of thyroid-guided palpation versus ultrasound-guided. The exclusion criteria were publications in the form of reviews, correspondences, editorials, and commentaries.

### Data extraction

Data extraction was conducted independently by two researchers. Data from studies that met the inclusion and exclusion criteria included the basic study characteristics. It also included the name of the principal researcher, type of study, place/country, year of publication, basic demographic characteristics of study subjects, sample size, thyroid FNAB examination technique, operator characteristics, blinding, and comparison results. The output was in the form of a descriptive table. The output was written in a two-by-two table form and displayed in sensitivity, specificity, DOR, and AUC.

### Study quality assessment

Two independent reviewers assessed study quality and risk of bias. Any discrepancies were resolved through consensus with an independent third party. The Quality Assessment of Diagnostic Accuracy Studies (QUADAS)-2 was used for the assessment of study quality, where each domain was assessed for risk of bias. QUADAS-2 consists of 4 main domains: patient selection, index testing, reference standards, flow, and time. Each domain has its risk of bias to assist in the suspicion of risk bias, including the cue questions.

### Statistical analysis

The statistical analysis in this study was performed using the RevMan software version 5.4 (Cochrane Collaboration, the Nordic Cochrane Center, Copenhagen) and Meta & Mada Package R version 4.0.3 [20, 21].

Heterogeneity was assessed using the snowballing method, I^2^ test, and Cochran’s test. A random-effects model selected if there were significant heterogeneity, whereas a fixed effect model will be selected if it was not significant. The results of data analysis were presented in the form of a forest plot and The Summary Receiver Operating Characteristic (SROC) curve when meta-analysis could be performed. The expected results were in the form of accuracy, sensitivity, and specificity along with the confidence interval, DOR, and AUC. A comparative analysis of the accuracy of each index test was conducted using a likelihood-ratio test, diagnostic meta-regression and Characteristic Receiver Operating Comparison test (ROC) using R application [21, 22].

### Publication bias

In this study, we constructed funnel plots of the Diagnostic Odds Ratio (DOR) to appraise whether there was a publication risk bias or not. This study has also been pre-registered in PROSPERO with the registration number CRD42020207291.

## Results

### Selection and identification of studies

From the three databases and grey literature used, 2517 articles were screened of which only 50 articles were potentially relevant based on the title and abstract. After studies were screened, 14 studies were included. Of the 14 studies that fulfilled the screening process, two studies conducted by Choong (2018) et al. and Guo (2015) et al. [5, 7, 18] had different outcome criteria from this study. In this study, true positive of malignancy is defined as indeterminant, suspicious malignant, and malignant, while in Choong et al. and Guo et al. studies, indeterminant were not included as true positive malignancy. However, a study by Taha et al. had a different number of subjects in the article than in the raw data. We attempted to send correspondence to the authors of these three studies to obtain raw data, no response was received until the paper was completed. As a result, only 11 articles were included in the meta-analysis, while the three articles were only included in the systematic review.

**Fig. 1:**
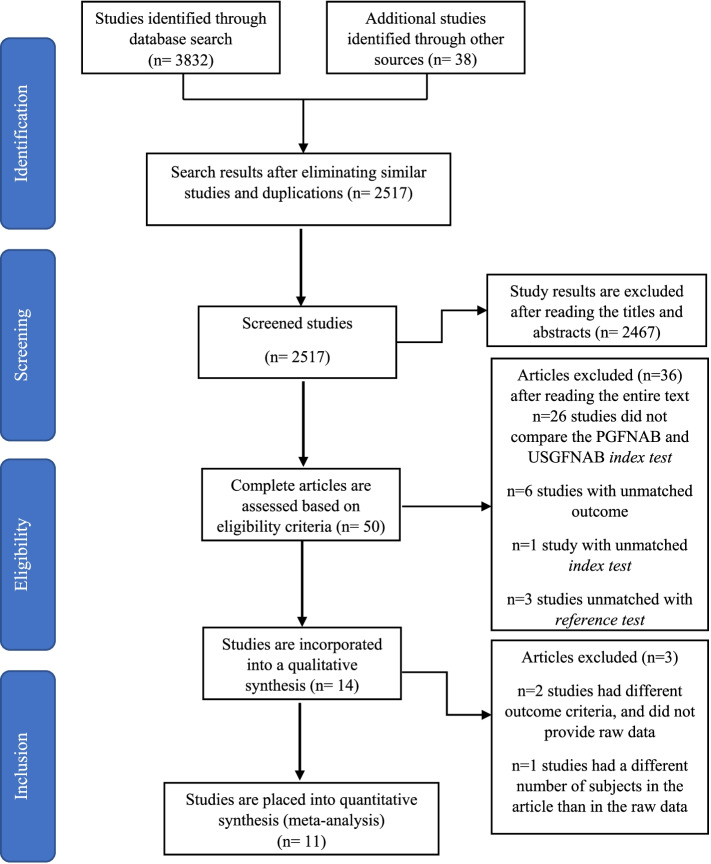
The scheme of PRISM

The total number of thyroid nodule patients in 14 studies was 6316 subjects. Among them, 3095 subjects used the PGFNAB method and 3221 subjects used USGFNAB. We evaluated 2382 subjects, of which 1128 subjects used PGFNAB and 1254 subjects used USGFNAB from those 11 articles [9–17, 19, 23]. The selection process is shown in Fig. 1.

Cohen’s Kappa coefficient on the title and abstract filter was 0.254, indicating that the level of agreement was minimal. The coefficient of Cohen’s Kappa on the eligibility criteria assessment was 0.957, suggesting that the level of agreement was good [24]. The difference in ratings occurred in 243 articles (out of 2517 articles) on the title and abstract screening, and in one article (out of 50 articles) on the full manuscript assessment. The differences in the ratings were resolved by discussion to reach a consensus between the 2 researchers.

### Characteristics of study

The studies had similar study population characteristics, study design, study site location, gold standard, and expected outcome. The detailed characteristics of the studies can be seen in Table 4. The publication year range of 14 studies were from 1994 [9] to 2020 [5]. Almost all studies had a cross-sectional design, except the study by Izquierdo et al. [15], a prospective cohort design. Each study compared the diagnostic accuracy of palpation-guided FNAB with ultrasound-guided FNAB. The gold-standard examination was essential in diagnostic study. This study used operative histopathology as the gold standard.

**Table 4:** The characteristics of the studies

No ID	First author (Publication year)	Country	Research Design	Technique	Subject age (Mean/median [year])	Sample size	Inadequate/ Non-diagnostic	benign	AUS, FLUS, FN (Indeterminant)	Malignancy suspicion, malignancy (Malignant)
1	Takashima et al. (1994) [9]	Japan	Cross Section	PGFNAB	53	34	0	12	0	22
				USGFNAB		99	0	32	0	67
2	Hatada et al. (1998) [11]	Japan	Cross Section	PGFNAB	54.7 ± 13.7	94	28	42	0	24
				USGFNAB	51.4 ± 16	72	12	37	0	23
3	Danese et al. (1998) [10]	Italy	Cross Section	PGFNAB	43	535	13	307	160	55
				USGFNAB		540	5	310	155	70
4	Carmeci et al. (1998) [12]	US	Cross Section	PGFNAB	49.2	47	6	13	7	21
				USGFNAB		17	2	1	4	10
5	Solymosi et al. (2001) [23]	Hungary	Cross Section	PGFNAB	N/A	354	37	197	0	120
				USGFNAB		420	40	302	0	78
6	Goudy et al. (2005) [13]	US	Cross Section	PGFNAB	55.4	20	2	12	2	4
				USGFNAB		11	0	4	6	1
7	Cesur et al. (2006) [14]	Turkey	Cross Section	PGFNAB	48.7 ± 13.5	26	8	11	3	4
				USGFNAB		26	4	14	4	4
8	Izquierdo et al. (2006) [15]	US	Perspective Cohort	PGFNAB	48.6 ± 17	23	0	11	5	7
				USGFNAB		5	0	1	3	1
9	Can et al. (2008) [16]	Turkey	Cross Section	PGFNAB	40 ± 12	18	3	12	1	2
				USGFNAB	44 ± 14	23	1	11	6	5
10	Krishnappa et al. (2013) [17]	India	Cross Section	PGFNAB	38.5	25	0	15	0	10
				USGFNAB		25	0	15	0	10
11	Guo et al. (2015) [18]	China	Cross Section	PGFNAB	44	101	2	–	27	72
				USGFNAB		388	8	–	57	323
12	Sharma et al. (2017) [19]	India	Cross Section	PGFNAB	36.4	49	0	38	7	4
				USGFNAB	36	80	0	74	3	3
13	Choong et al. (2018) [7]	US	Cross Section	PGFNAB	51.5	1199	60	725	285	129
				USGFNAB	53.6	1123	50	639	286	148
14	Taha et al. (2020) [5]	Qatar	Cross Section	PGFNAB	46.3 ± 11.7	570	98	398	52	140
				USGFNAB		392	51	215	58	146

Penjelasan dari AUS, FLUS, FN

In general, the literature was relatively heterogeneous. There was heterogeneity in the results of each study; corresponding calculations of Cochran’s Q test were *p* < 0.05 in both the PGFNAB and USGFNAB methods.

Moreover, there were varieties of the mean patient ages, gender, places, and countries (see Table 4). The mean patient age varied in each study from 36 to 55.4 years, with the female sex being the most involved in these 14 studies. The studies were performed in the radiology department, clinical pathology, thyroid clinic and surgery department. This meta-analysis study represents various countries in the world such as Japan, Italy, America, Hungary, Turkey, India, and Qatar [9–17, 19, 23].

They also varied in operator, size of the needle used, and nodule size. The biopsy needles used varied from 21 to 25 gauge sizes. The ultrasound used in each study used 5 Mhz and 7.5 Mhz transducers to visualize the needle during aspiration of the thyroid nodule. Almost all nodules in the PGFNAB method were more than 1 cm in size and could be palpated with a mean value ranging from 1.22 to 2.85 cm. The size of the nodules on the USGFNAB method were diverse. Some nodules were less than 1 cm in size, difficult to palpate, not palpable, and more than 1 cm with a mean value ranging from 1.17 to 2.88 cm [9–17, 19, 23]. Three studies did not include nodule sizes, such as the studies by Krishnappa et al. [17], Sharma et al. [19], and Solymossy et al. [23].

### Quality assessment

**Fig. 2:**
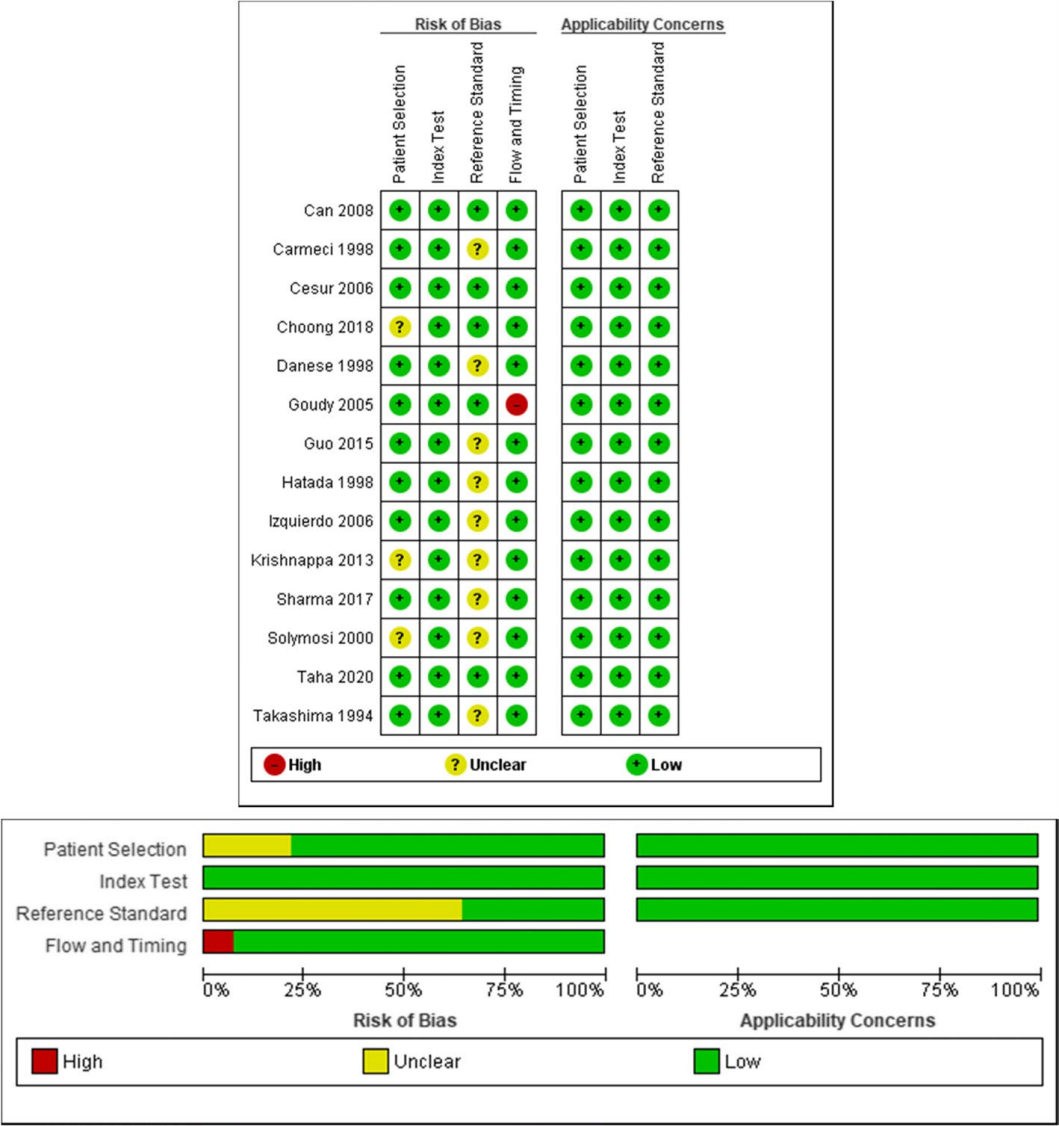
Assessment of Quality of Studies and Risk Bias with QUADAS-2

The assessment for each study using the QUADAS-2 tool was shown in Fig. 2 with the details in Table 5. In general, the risk of bias assessment in each study was mild to moderate, and the quality of each study in this systematic review was good. The original studies from Can et al. [16], Cesur et al. [14], and Taha et al. [5] had the least risk of bias. Of the 14 studies, several other studies had questionable aspects in the components assessed in QUADAS-2.

**Table 5:** Detailed assessment of quality of studies and risk bias with QUADAS-2

Assessment	Details
Patient Selection	If the study explained that the USGFNAB and PGFNAB samples were taken sequentially or randomly, the control study design was avoided, and that unnecessary exclusion was avoided, it was given a positive (+) sign or low bias. If the sample entered falls under the research question, the applicability is given a positive sign (+).
Index Test	If the study explained that the PGFNAB and USGFNAB tests were performed without knowing the histopathological results and that the threshold had been determined previously, it was given a positive sign (+) or low bias. If the index test is performed and the interpretation falls under the research questions, the applicability is given a positive sign (+).
Reference Standard	If the study explains the gold standard according to the standard and researchers interpret it without first knowing the index test results, they are given a positive sign (+) or low bias. If the study does not explain the type of gold standard or if the researcher interprets the gold standard without first knowing the index test results, the study is unclear. If the target conditions are being studied, the applicability is given a positive sign (+).
Flow and Timing	When describing the PGFNAB/USGFNAB interval with operative histopathology, the study was given a positive (+) sign or low bias, all patients were given the gold standard, and all patients were included in the analysis. Not all of the patients in Goudy’s study were included in the analysis.

### Diagnostic accuracy and inadequacy

**Fig. 3:**
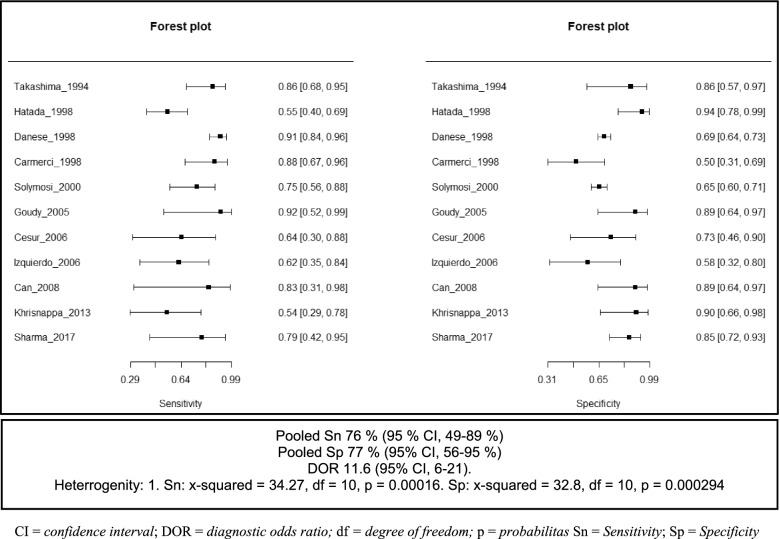
Forest Plot of sensitivity and specificity of PGFNAB method

The diagnostic accuracy with raw data values of 2 × 2 tables and the outcomes of each study can be seen in Tables 6 and 7, respectively. Of the 11 studies analyzed, the sensitivity of the PGFNAB method in diagnosing thyroid cancer was reported being between 55 to 100%, with a pooled sensitivity calculation of 76% (95% CI, 64–84%). The forest plot of PGFNAB were shown in Fig. 3.

**Table 6:** The results of diagnostic accuracy from each study

Study (year)	Technique	Sample size	Diagnosis of cancer based on histopathology						
			Sensitivity	Specificity	PPV	NPV	LR (+)	LR (−)	Accuracy
Takashima et al. (1994) [9]	PGFNAB	34	87.50	90.00	95.45	75.00	8.75	0.14	88.2
	USGFNAB	99	95.52	90.63	95.52	90.63	10.19	0.05	93.9
Hatada et al. (1998) [11]	PGFNAB	66	54.76	95.83	95.83	54.76	13.14	0.47	69.7
	USGFNAB	60	66.7	96.3	95.7	70.3	18.0	0.3	80.0
Danese et al. (1998) [10]	PGFNAB	522	91.9	68.8	36.7	97.7	2.9	0.1	72.6
	USGFNAB	535	97.1	70.9	44.0	99.0	3.3	0.0	75.9
Carmeci et al. (1998) [12]	PGFNAB	41	89.5	50.0	60.7	84.6	1.8	0.2	68.3
	USGFNAB	15	100.0	20.0	71.4	100.0	1.3	0.0	73.3
Solymosi et al. (2001) [23]	PGFNAB	317	76.0	65.4	15.8	97.0	2.2	0.4	66.2
	USGFNAB	380	92.1	70.2	25.5	98.8	3.1	0.1	72.4
Goudy et al. (2005) [13]	PGFNAB	18	100.0	92.3	83.3	100.0	13.0	0.0	94.4
	USGFNAB	11	100.0	44.4	28.6	100.0	1.8	0.0	54.5
Cesur et al. (2006) [14]	PGFNAB	18	66.7	75.0	57.1	81.8	2.7	0.4	72.2
	USGFNAB	22	85.7	86.7	75.0	92.9	6.4	0.2	86.4
Izquierdo et al. (2006) [15]	PGFNAB	23	63.6	58.3	58.3	63.6	1.5	0.6	60.9
	USGFNAB	5	100.0	50.0	75.0	100.0	2.0	0.0	80.0
Can et al. (2008) [16]	PGFNAB	15	100.0	92.3	66.7	100.0	13.0	0.0	93.3
	USGFNAB	22	100.0	78.6	72.7	100.0	4.7	0.0	86.4
Krishnappa et al. (2013) [17]	PGFNAB	25	54.5	92.9	85.7	72.2	7.6	0.5	76.0
	USGFNAB	25	81.8	92.9	90.0	86.7	11.5	0.2	88.0
Guo et al. (2015) [18]	PGFNAB	99	93.4	95.7	98.6	81.5	21.5	0.1	93.9
	USGFNAB	380	90.4	66.7	96.3	42.1	2.7	0.1	88.2
Sharma et al. (2017) [19]	PGFNAB	49	83.3	86.0	45.5	97.4	6.0	0.2	85.7
	USGFNAB	80	75.0	96.1	50.0	98.6	19.0	0.3	95.0
Choong et al. (2018) [7]	PGFNAB	355	86.2	100	100	97.38	0.0	0.14	97.7
	USGFNAB	228	85.71	99.4	98.2	94.8	141	0.14	95.6
Taha et al. (2020) [5]	PGFNAB	570	52.3	94.3	73.1	87.0	9.2	0.5	84.7
	USGFNAB	392	69.7	91.1	75.3	88.6	7.8	0.3	85.1

*LR* Likelihood ratio

**Table 7:** The diagnostic results of each study included in the meta-analysis

Study (year)	Technique	Sample size	*True Positives*	*False Positives*	*False Negatives*	*True Negatives*
Takashima et al. (1994) [9]	PGFNAB	34	21	1	3	9
	USGFNAB	99	64	3	3	29
Hatada et al. (1998) [11]	PGFNAB	66	23	1	19	23
	USGFNAB	60	22	1	11	26
Danese et al. (1998) [10]	PGFNAB	522	79	136	7	300
	USGFNAB	535	99	126	3	307
Carmeci et al. (1998) [12]	PGFNAB	41	17	11	2	11
	USGFNAB	15	10	4	0	1
Solymosi et al. (2001) [23]	PGFNAB	317	19	101	6	191
	USGFNAB	380	35	102	3	240
Goudy et al. (2005) [13]	PGFNAB	18	5	1	0	12
	USGFNAB	11	2	5	0	4
Cesur et al. (2006) [14]	PGFNAB	18	4	3	2	9
	USGFNAB	22	6	2	1	13
Izquierdo et al. (2006) [15]	PGFNAB	23	7	5	4	7
	USGFNAB	5	3	1	0	1
Can et al. (2008) [16]	PGFNAB	15	2	1	0	12
	USGFNAB	22	8	3	0	11
Krishnappa et al. (2013) [17]	PGFNAB	25	6	1	5	13
	USGFNAB	25	9	1	2	13
Sharma et al. (2017) [19]	PGFNAB	49	5	6	1	37
	USGFNAB	80	3	3	1	73

Meanwhile, the specificity of PGFNAB in diagnosing thyroid cancer ranged from 50 to 96%, with a pooled specificity of 77% (95% CI, 56–95%). The pooled Diagnostic Odds Ratio (DOR) was 11.6 (95% CI, 6–21) and the Area Under Curve (AUC) was 0,827.

**Fig. 4:**
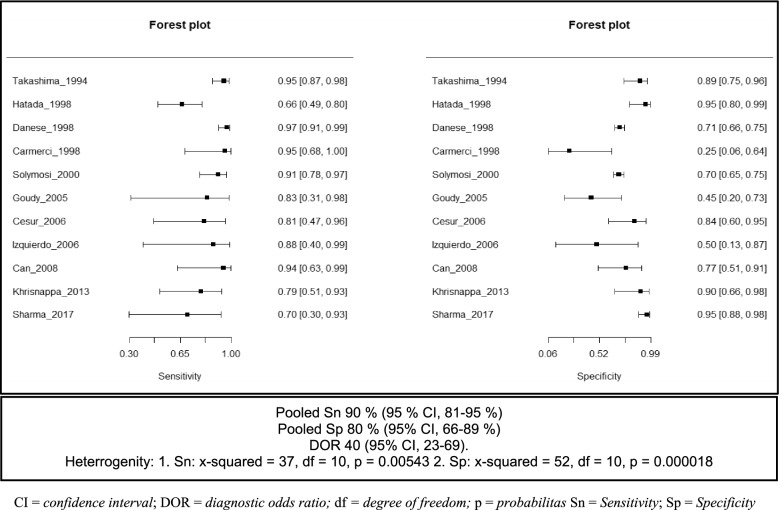
Forest Plot sensitivity and specificity of USGFNAB method

The sensitivity of USGFNAB in diagnosing thyroid cancer was in the range of 67 to 100%, with a pooled sensitivity calculation of 90% (95% CI, 49–89%). The specificity of PGFNAB in diagnosing thyroid cancer was in the range of 50 to 96%, with a pooled specificity of 80% (95% CI, 56–95). The pooled Diagnostic Odds Ratio (DOR) was 40 (95% CI, 23–69) and the Area Under Curve (AUC) was 0.92. The forest plot of USGFNAB is shown in Fig. 4.

**Fig. 5:**
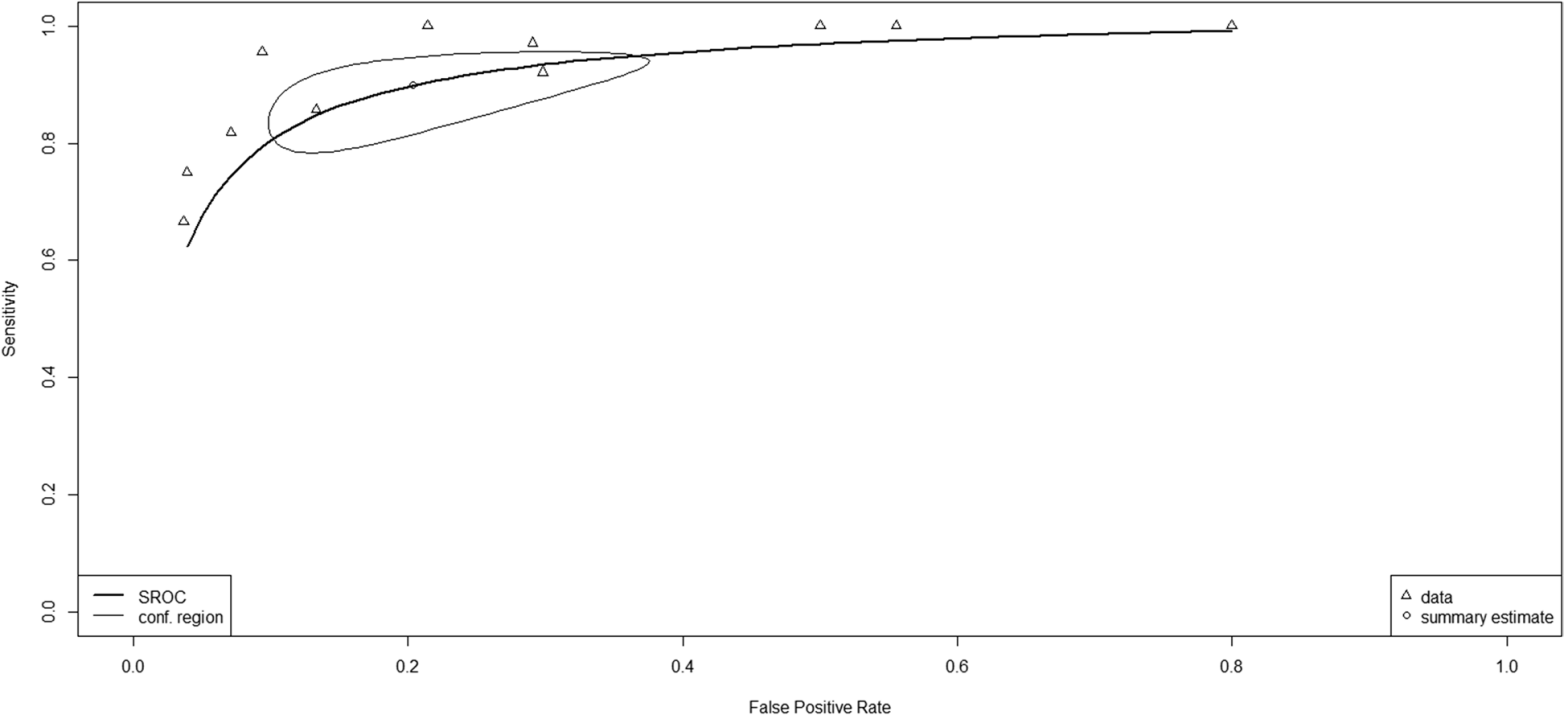
SROC curve comparison of PGFNAB vs USGFNAB for the diagnosis of thyroid cancer

From the two index tests, a comparison test was conducted to determine diagnosis accuracy by performing the likelihood ratio test and getting a chi-square result of 6.28, *P* = 0.0043. This suggests a significant differences between the two index tests. Subsequently, a diagnostic meta-regression was performed. This test assessed the sensitivity transformation and false positive rate transformation with the regression coefficient value of Tsens 0.99 (*p* = 0.023) and Tfpr − 0.120, (*p* = 0.760). The details of the comparison and SROC curve of PGFNAB vs USGFNAB can be seen in Table 8 and Fig. 5.

**Table 8:** Summary of comparison of PGFNAB vs USGFNAB diagnostic accuracy

The number of studies	Method	Number of patient	Sensitivity	Specificity	AUC	Test^c^
11	PGFNAB	1128	76%	77%	0.827	
	USGFNAB	1254	90%	80%	0.92	
	(95% CI); *P*-*value*		Tsens^a^ = 0.99 (0.14,1.84), *P* = 0.023	Tfpr^a^ = −0.120 (−0.89,0.65) *P* = 0.760	Diff^b^ = 0.093 *P* = 0.000023	*ChiSquared* = *6*.28 *P* = 0.043

*AUC* Area Under Curve, *CI* Confidence interval, *Diff* Difference, *Tfpr* Transformed false positive rate, *Tsens* Transformed sensitivity

^a^Comparison of differences in sensitivity and specificity of PGFNAB and USGFNAB using *diagnostic meta regression*

^b^Comparison of the differences in AUC from PGFNAB and USGFNAB using the ROC comparison test

^c^Comparison of the differences in sensitivity and specificity between PGFNAB and USGFNAB using the likelihood-ratio test

The rate of inadequacy varied between the two methods. For the PGFNAB method, the inadequacy rate ranged from 2 to 32%, with a mean value of 14.6%, while the inadequacy rate of USGFNAB ranged from 0 to 21.5% with a mean value of 9%. There was a significant difference with a *p* value < 0.0001.

### Publication bias

**Fig. 6:**
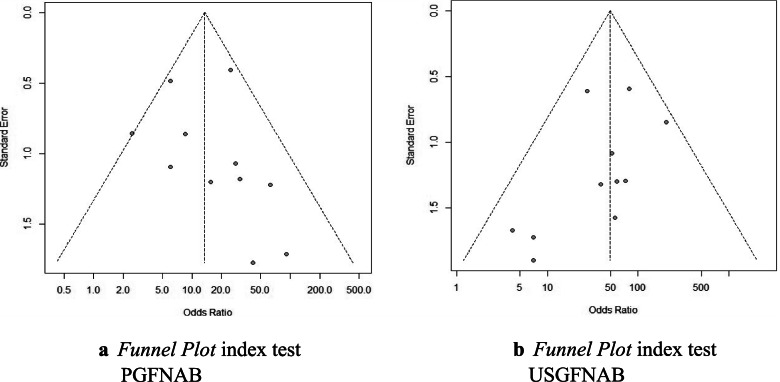
**a***Funnel Plot* index test PGFNAB. **b***Funnel Plot* index test USGFNAB

A funnel plot of the diagnostic value of the ratio was made to assess a publication risk of bias in this systematic review. The results of the funnel plot of PGFNAB and USGFNAB can be seen in Fig. 6a and b respectively. In the funnel plot, it was relatively symmetrical in both the PGFNAB and USGFNAB funnel plot groups. These figures suggested that there may be a minimal risk of publication bias in this systematic review.

## Discussion

In this meta-analysis, we evaluated the diagnostic accuracy of PGFNAB and USGFNAB in diagnosing thyroid nodule malignancy. The results of the USGFNAB index test showed that the values for pooled sensitivity, pooled specificity, DOR, and AUC were 90, 80%, 40 and 0.92 respectively and had an estimated point on the SROC Curve in the upper left (see Fig. 5). These results indicated that the USGFNAB index test had excellent diagnostic accuracy. PGFNAB index test had lower results for pooled sensitivity, pooled specificity, DOR, and AUC than USGFNAB, namely 76, 77%, 11, and 0.827, respectively.

The positive Tsens regression coefficient suggests that the USGFNAB sensitivity was better than the PGFNAB, and a *p* value < 0.05 indicated that the result was statistically significant. The regression coefficient for Tfpr was negative, suggesting that the specificity of PGFNAB was better than USGFNAB, yet these results were not statistically significant. Relating to AUC value significance, a difference of 0.093 (*p* = 0.000023) was found. The comparison of the SROC curve image showed that the estimation points of the two curves were very distinct from the spheres or the CI values that were slightly intersected (see Fig. 5), suggesting a significant difference between the two index tests.

Meta-analyzes assessing the accuracy of the PGFNAB and USGFNAB diagnoses had previously been performed. Two meta-analyses evaluating the accuracy of the diagnosis of FNAB in the thyroid was noted. First, Ospina et al. [4] conducted a meta-analysis pertaining the accuracy of the diagnosis of USGFNAB in thyroid nodules but did not compare it to PGFNAB. Second, Matz et al. [6] on this meta-analysis assessed the comparison between USGFNAB and PGFNAB. The results of this meta-analysis were consistent with the results of the study by Matz et al., where the pooled sensitivity value of USGFNAB was higher than that of PGFNAB [0.91 (CI = 0.82, 1.0) and 0.79 (CI = 0.69, 0.85), respectively]. However, the pooled specificity values were slightly higher for USGFNAB than in PGFNAB [0.77 (CI = 0.69, 0.85) and 0.73 (CI = 0.64, 0.81), respectively]. Matz et al. conducted a comparison between the two tests using the SROC curve [6]. Yet, no comparison using the diagnostic meta-regression and likelihood-ratio test was used, unlike this meta-analysis.

A study conducted by Taha et al. [5] showed that the sensitivity value of USGFNAB was greater than that of PGFNAB, namely 69 and 52%, respectively. Meanwhile, the PGFNAB specificity value was slightly higher than USGFNAB at 94 and 91%, respectively [5]. However, this study was not included in this meta-analysis because the raw data displayed between the number of tests performed and those described was not suitable.

Studies by Choong et al. [7] and Guo et al. [18], were not included in this meta-analysis study due to differences in the primary criteria used in the 2 × 2 table. The results of the studies were different from the majority of previous studies. In these studies, the sensitivity and specificity values of PGFNAB were greater than USGFNAB. In the study by Choong et al., the sensitivity and specificity values were 86% vs 85.5 and 100% vs 99%, respectively [7]. In the study by Guo et al., the sensitivity and specificity values were 93% vs 90 and 96% vs 67%, respectively [18].

The benign criteria was used for indeterminacy/ AUS/ FLUS/ FN, suspicion of malignancy as the criteria for malignancy. It aimed to create a 2 × 2 table and determine true positive, false positive, true negative and false negative values in the index test column. For the gold-standard column, the histo-pathological results of the surgery were divided into benign and malignant. In some studies, indeterminant groups were classified as benign, and some were categorized as malignant. If it is included in the malignant criteria in the independent group, it can increase the false positive number on the result [6, 16].

Some previous studies suggest that USGFNAB is obviously preferable in patients with non-palpable or difficult to palpate nodule, predominantly cystic nodules with a small solid component and non-diagnostic PGFNAB, whether USGFNAB should be preferentially used for all palpable nodules is not clear.^1,14^ However, in this meta-analysis, the size of the nodules in the PGFNAB method were almost all larger than 1 cm and can be palpated. In the USGFNAB method, there were nodules less than 1 cm, nodules difficult to palpate, nodules not palpable and nodules greater than 1 cm. Therefore, the results of this meta-analysis found that USGFNAB is preferable for all palpable and non-palpable nodules.

The inadequacy number of PGFNAB method and the USGFNAB method were 14.6 and 9%, respectively. From these results, there was a significant difference between the two with a *P* = < 0.0001, suggesting that the USGFNAB method had better results compared to PGFNAB. These results were consistent with the study by Matz et al., in which inadequacy rate of PGFNAB was 14.7% and USGFNAB was 8.4% [6]. Moreover, in a meta-analysis carried out by Gharib et al, in which more than 18,000 cases were evaluated, the inadequacy rates of FNAB was 17% [8].

The occurrence of inadequate material after a biopsy may be caused by several factors including: nodule size; number of aspiration times during FNAB; operator factors; and the results’ definition, which were inadequate in each study [14, 16]. Some studies have suggested that the adequate rate of biopsy results increased with increasing nodule size [13, 14, 25]. Aspiration during FNAB was recommended 2–4 times aspiration per one nodule [26–29].

The quality of the main outcome of this meta-analysis was assessed based on the Grading of Recommendations Assessment, Development and Evaluation (GRADE) approach. It included the risk of bias, imprecision, inconsistency, indirectness, and publication bias. Each section was assessed, one-point reduction for any significant findings and two-points reduction for very significant findings or no serious findings (not reduced). The results of the quality assessment were divided into high, moderate, low, and very low. The results of the assessment can be seen in Table 9.

**Table 9:** Summary of findings for the diagnostic accuracy of PGFNAB vs USGFNAB

Outcome	Number of subjects (number of studies)	***Pooled effect estimates***	Quality of evidence (GRADE)	Summary of evidence quality
All ages	2382 (11 studies)	PGFNAB: Sn 76% (95% CI, 49–89%) Sp 77% (95% CI, 56–95%) AUC = 0.827	⨁⨁⨁◯ There is *heterogeneity*	Sufficient
		USGFNAB: Sn 90% (95% CI, 81–95%) Sp 80% (95% CI, 66–89%) AUC = 0.92		

Description related to the evidence quality

⨁⨁⨁⨁: High. The authors are confident that the effect obtained in this meta-analysis is an effect which accurately happened

⨁⨁⨁◯: Sufficient. The authors are reasonably confident that the effect obtained in this meta-analysis is an approximate actual effect, but there is still a possibility that there may be a substantial effect difference happened

⨁⨁◯◯: Low. The authors have limited confidence in the effect obtained in this meta-analysis. The actual effect could be significantly different from the effect obtained in this meta-analysis

⨁◯◯◯: Very low. The authors are not sure of the effect obtained. This meta-analysis might have the same as the actual effect

The weaknesses of this study is that several studies did not display the results entirely, so that complete data cannot be obtained to make 2 by 2 contigency tables according to the research criteria. Therefore, no intersection point for measuring the output parameters was included in this meta-analysis. Also, heterogeneity is still present in this meta-analysis.

## Conclusion

The diagnostic accuracy (sensitivity and specificity) of USGFNAB is significantly higher than PGFNAB in diagnosing thyroid cancer with palpable or nonpalpable nodules. The quality of the studies reviewed in this study are good. As a result, the quality of output evidence based on GRADE is sufficient. If it is accessible, USGFNAB is more recommended as a diagnostic tool for thyroid nodules.

## Data Availability

The datasets used and/or analysed during the current study available from the corresponding author and co-author (TJET, HC) on reasonable request.

## References

[1] Haugen BR, Alexander EK, Bible KC, Doherty GM, Mandel SJ, Nikiforov YE (2016). 2015 American Thyroid Association management guidelines for adult patients with thyroid nodules and differentiated thyroid cancer: the American Thyroid Association guidelines task force on thyroid nodules and differentiated thyroid cancer. Thyroid.

[2] Saksono D, Soewondo P, Subekti I, Soebardi S, Darmowidjoyo B, Purnamasari D, Tarigan TJE, Wisnu WTD (2018). Petunjuk praktis pengelolaan nodul tiroid.

[3] Pemayun T (2016). Current diagnosis and management of thyroid nodules. Acta Med Indones-Indones J Intern Med..

[4] Ospina NS, Brito JP, Maraka S, Espinosa de Ycaza AE, Rodriguez-Gutierrez R, Gionfriddo MR (2016). Diagnostic accuracy of ultrasound-guided fine needle aspiration biopsy for thyroid malignancy: systematic review and meta-analysis. Endocrine.

[5] Taha I, Al-Thani H, El-Menyar A, Asim M, Al-Sulaiti M, Tabeb A (2020). Diagnostic accuracy of preoperative palpation- versus ultrasound-guided thyroid fine needle aspiration cytology: an observational study. Postgrad Med.

[6] Matz J, Abdolell M, Hayden J, Nasser J (2014). A systematic review and meta-analysis of palpation versus ultrasound-guided fine needle aspiration of thyroid nodules. Dalhousie Med J.

[7] Choong KC, Khiyami A, Tamarkin SW, McHenry CR (2018). Fine-needle aspiration biopsy of thyroid nodules: is routine ultrasound-guidance necessary?. Surgery.

[8] Gharib H, Papini E, Valcavi R, Baskin J, Crescenzi A, Dottorini ME (2006). American association of clinical endocrinologists and associazione medici endocrinologi medical guidelines for clinical practice for the diagnosis and management of thyroid nodules. Endocr Pract.

[9] Takashima S, Fukuda H, Kobayashi T (1994). Thyroid nodules: clinical effect of ultrasound-guided fine-needle aspiration biopsy. J Clin Ultrasound.

[10] Danese D, Sciacchitano S, Farsetti A, Andreoli M, Pontecorvi A (1998). Diagnostic accuracy of conventional versus sonography-guided fine-needle aspiration biopsy of thyroid nodules. Thyroid.

[11] Hatada T, Okada K, Ishii H, Ichii S, Utsunomiya J, Hyogo. (1998). Evaluation of ultrasound-guided fine-needle aspiration biopsy for thyroid nodules. Am J Surg.

[12] Carmeci C, Brooke Jeffrey R, McDougall IR, Nowels KW, Weigel RJ, Jeffrey RB (1998). Ultrasound-guided fine-needle aspiration biopsy of thyroid masses. Thyroid.

[13] Goudy SL, Flynn MB (2005). Diagnostic accuracy of palpation-guided and image-guided fine-needle aspiration biopsy of the thyroid. Ear Nose Throat J.

[14] Cesur M, Corapcioglu D, Bulut S, Gursoy A, Yilmaz AE, Erdogan N (2006). Comparison of palpation-guided fine-needle aspiration biopsy to ultrasound-guided fine-needle aspiration biopsy in the evaluation of thyroid nodules. Thyroid.

[15] Izquierdo R, Arekat MR, Knudson PE, Kartun KF, Khurana K, Kort K (2006). Comparison of palpation-guided versus ultrasound-guided fine-needle aspiration biopsies of thyroid nodules in an outpatient endocrinology practice. Endocr Pract.

[16] Can AS, Peker K (2008). Comparison of palpation-versus ultrasound-guided fine-needle aspiration biopsies in the evaluation of thyroid nodules. BMC Res Notes.

[17] Krishnappa P, Ramakrishnappa S, Kulkarni MH (2013). Comparison of free hand versus ultrasound-guided fine needle aspiration of thyroid with histopathological correlation. J Environ Pathol Toxicol Oncol.

[18] Guo HQ, Zhang ZH, Zhao H, Niu LJ, Chang Q, Pan QJ (2015). Factors influencing the reliability of thyroid fine-needle aspiration: analysis of thyroid nodule size, guidance mode for aspiration and preparation method. Acta Cytol.

[19] Sharma M, Mahore S (2017). A comparison of the diagnostic efficiency of guided fine needle aspiration cytology versus conventional fine needle aspiration cytology of the thyroid. Indian J Otolaryngol Head Neck Surg.

[20] Schwarzer G (2021). General package for meta-analysis.

[21] Doebler P (2020). R Package mada “meta-analysis of diagnostic accuracy”.

[22] Hanley JA, McNeil BJ (1982). The meaning and use of the area under a receiver operating characteristic (ROC) curve. Radiology.

[23] Solymosi T, Toth GL, Bodo M (2001). Diagnostic accuracy of fine needle aspiration cytology of the thyroid: impact of ultrasonography and ultrasonographically guided aspiration. Acta Cytol.

[24] McHugh ML (2012). Lessons in biostatistics interrater reliability: the kappa statistic. Biochem Med.

[25] Baloch ZW, Livolsi VA (2004). Symposium article fine-needle aspiration of thyroid nodules. Endocr Pract.

[26] Cheung YS, Poon CM, Mak SM, Suen MWM, Leong HT (2007). Fine-needle aspiration cytology of thyroid nodules - how well are we doing?. Hong Kong Med J.

[27] Di Fermo F, Sforza N, Rosmarin M, Morosan Allo Y, Parisi C, Santamaria J (2020). Comparison of different systems of ultrasound (US) risk stratification for malignancy in elderly patients with thyroid nodules. Real world experience. Endocrine.

[28] Rausch P, Nowels K, Jeffrey RB (2001). Ultrasonographically guided thyroid biopsy: a review with emphasis on technique. J Ultrasound Med.

[29] Baloch ZW, Tam D, Langer J, Mandel S, LiVolsi VA, Gupta PK (2000). Ultrasound-guided fine-needle aspiration biopsy of the thyroid: role of on-site assessment and multiple cytologic preparations. Diagn Cytopathol.

